# Survival Benefits With Artificial Liver Support System for Acute-on-Chronic Liver Failure: A Time Series-Based Meta-Analysis

**DOI:** 10.1097/MD.0000000000002506

**Published:** 2016-01-22

**Authors:** Yi Shen, Xu-Lin Wang, Bin Wang, Jian-Guo Shao, Yan-Mei Liu, Yan Qin, Lu-Jun Wang, Gang Qin

**Affiliations:** From the Department of Epidemiology and Medical Statistics (YS, X-LW, Y-ML); Center for Liver Diseases, Nantong Third People's Hospital, Nantong University, China (BW, J-GS, L-JW, GQ); and Department of Internal Medicine, Singapore General Hospital, Singapore (YQ).

## Abstract

The artificial liver support system (ALSS) offers the potential to improve the prognosis of patients with acute-on-chronic liver failure (ACLF). However, the literature has been inconsistent on its survival benefits. We aimed to conduct a time series-based meta-analysis of randomized clinical trials (RCTs) and observational studies which examined differences in mortality in ACLF patients treated with ALSS or not.

MEDLINE, EMBASE, OVID, and COCHRANE library database were systemically searched up to December 2014. Quality of included studies was evaluated using the Jadad score. The outcome measure was mortality at different follow-up endpoints. Odds ratios (ORs) and survival curve data were pooled for analysis.

Ten studies, 7 RCTs, and 3 controlled cohorts were enrolled, involving a total of 1682 ACLF patients, among whom 842 were treated with ALSS. ALSS was found to reduce the risk of short-term (1-month and 3-month) mortality for patients with ACLF by nearly 30%. Randomized trials and observational studies provided good internal and external validity respectively. The combined Kaplan–Meier curves showed a consistent pattern of findings. Meta-analysis also suggested that ALSS might reduce medium-term (6-month and 1-year) mortality risk by 30% and long-term (3-year) mortality risk by 50% in ACLF patients.

ALSS therapy could reduce short-term mortality in patients with ACLF. Meanwhile, its impacts on medium- and long-term survival seem to be promising but remained inconclusive. Clinical utility of this system for survival benefit may be implied.

## Abstract 2

Supplemental Digital Content is available in the text

## INTRODUCTION

Acute-on-chronic failure (ACLF), defined as an acute deterioration of known or unknown chronic liver disease, is a serious medical ailment, and its incidence is increasing with the high prevalence of alcoholic liver disease (ALD), nonalcoholic fatty liver disease (NAFLD), hepatitis C virus (HCV) infection in developed countries, and hepatitis B virus (HBV) infection in China.^1^ ACLF is characterized by jaundice, coagulopathy, hepatic encephalopathy (HE), and high incidence of short-term (28-day) mortality of 30% to 40%.^2^ The outcome of standard medical treatment for such patients is poor. Liver transplantation (LT) represents an adequate treatment, but remains limited due to organ scarcity. Hence, there is an unmet medical need for new therapeutic options.

The artificial liver support system (ALSS) was first applied to treat acute liver failure in 1970s with the attempt to replace the detoxification functions of the liver. Later, several methods have been added to the ALSS inventory. Plasma exchange (PE) represents a complete detoxification but is mainly limited by increased risk of allergies and infections due to exposure to exogenous plasma. Albumin dialysis allows the removal of albumin-bound toxins and soluble toxins, with external albumin in MARS (molecular adsorbent recirculating system) or the patient's own albumin in FPSA (fractionated plasma separation and adsorption,). Although some clinical trials have demonstrated the beneficial effects of ALSS on HE^3,4^ and hepatorenal syndrome (HRS),^5^ studies on survival outcomes are controversial. Positive association of ALSS with short-term or long-term survival was suggested by several studies.^6,7^ In the meantime, inconclusive evidence, such as negative association, no association, or positive but not significant association, was suggested by other studies.^3,5,8^ Moreover, previous meta-analyses have revealed less consistent results.^9–13^ The seemingly conflicting findings may result from quite a few factors such as variable follow-up periods, variable ALSS methods, and variation across the populations. Thus, it is imperative to perform a more comprehensive and systematic meta^-^analysis to re-evaluate the effects of ALSS on the survival outcomes of patients with ACLF.

Quantitative meta-analysis which combines information from the same endpoint results may be a rational approach to estimate an overall effect and to investigate sources of heterogeneity. However, some articles showed survival data indirectly with Kaplan–Meier curves and did not provide detailed data for each endpoint. With the development of software GetData Graph Digitizer 2.24 (http://getdata-graph-digitizer.com/), the digitization and extraction of the data have become possible.^14^ Thus, using GetData software, we could extract data at specific time points and bring the observation periods into accord.

In this study, we conducted a time series-based meta-analysis to evaluate the efficacy and safety of ALSS for ACLF. The analyses were mainly based on randomized trials. A few nonrandomized studies were also included for explorative analyses.

## METHODS

### Data Sources

Preferred Reporting Items for Systematic Reviews and Meta-analysis (PRISMA) guidelines were followed to conduct the present meta-analysis.^15^ We searched MEDLINE, EMBASE, OVID, and COCHRANE library database (up to December 2014) using the following terms: (“acute-on-chronic liver failure” or “liver failure, chronic” or “liver failure” or “hepatic failure” or “end-stage liver disease”) and (“artificial liver” or “liver, artificial” or “liver support system” or “extracorporeal liver”). All data of this study were from previous published studies; thus no ethical approval and patient consent are required.

### Study Selection

Two coauthors (X-LW and BW) screened the title and abstracts of retrieved citations. Full texts of those citations were assessed according to the following inclusion criteria:

1. Randomized clinical trials (RCTs) or observational studies involved patients with objective diagnosis of ACLF.
2. Interventions (treatment groups) included ALSS, whereas the comparison interventions (control groups) adopted standard medical therapy (SMT).
3. The survival outcome data provided in articles was sufficient and had a critical endpoint, follow-up period ≥ 28 days.
4. Published in English.

### Data Extraction

The data from the included articles were extracted independently by 2 coauthors (XLW and YS). Disagreements were resolved through discussion or consultation with a third author (GQ or LJW). The following characteristics were collected in each study: first author, year of publication, country of origin, study design, recruitment period, duration of follow-up, number of research centers, definition of ACLF, the sample size of each group, demographic and clinical information of the participants, the incidence of adverse events, characteristics of the ALSS used (including the method, number of sessions, duration per session, blood flow rate, etc). Serious adverse events (SAEs) were defined by the level of intervention as registered in each study. If the articles showed survival data indirectly with Kaplan–Meier curves, the GetData Graph Digitizer was applied to digitize and extract the time-specific data.

The quality of citation was assessed according to the Jadad score.^16^ The outcome measure was mortality at different follow-up endpoints. Short-, medium-, and long-term mortality measurements were assessed from reported follow-up periods of 1 to 3 months, 6 months to 1 year, and 3 to 5 years, respectively.

### Statistical Analysis

The odds ratio (OR) with 95% confidence intervals (95% CI) were calculated for dichotomous outcomes. Heterogeneity across studies was assessed with the Cochrane Q statistics the *I*^*2*^ test. An *I*^*2*^ value of >50% was considered substantial heterogeneity and the random-effect model was conducted; otherwise, the fixed-effect model was used. Publication bias was evaluated with the rank correlation test (Begg's test), the regression asymmetry test (Egger's test), and the funnel plots visually.^17^ Meta-regression was performed to investigate the sources of heterogeneity in the included studies. Subgroup analysis was performed according to the study design (randomized or nonrandomized studies). Moreover, a sensitivity analysis was performed using a 1-study-removed analysis. Kaplan–Meier estimates of survival data (where estimable) comparing ALSS with control groups were analyzed using an unadjusted log-rank test. For categorical variables, data were analyzed using the χ^2^ and Fisher's exact tests as appropriate.

All analyses were performed with Stata version 13.0 (StataCorp, TX). Statistically significant findings were defined as those with a *P* value ≤ 0.05.

## RESULTS

### Literature Search

**FIGURE 1:**
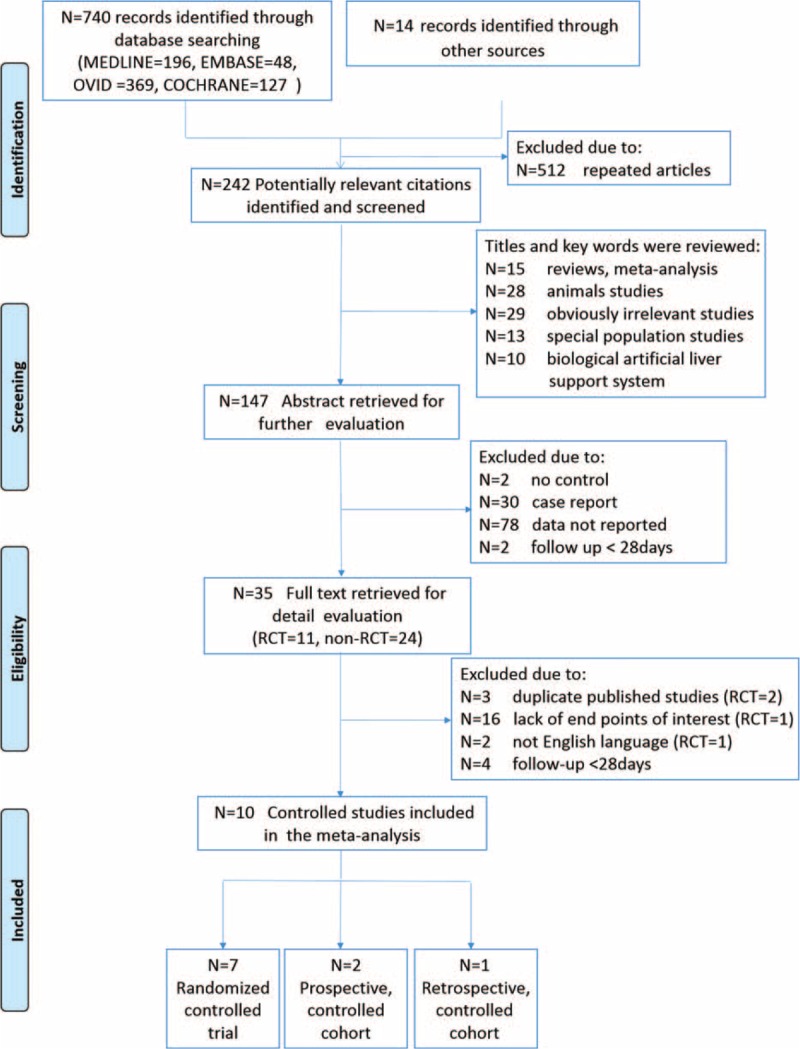
Flowchart of study selection.

Figure 1 described the search procedures. In brief, a total of 242 potentially relevant articles were identified through online database search. After reviewing the titles and abstracts, 95 and 112 articles were excluded respectively. For the 35 full-text articles retrieved, we excluded 25 articles due to duplicate publications, lack of relevant subpopulations, incomplete data, or non-English language. Finally, 10 articles, published between 2000 and 2014, were eligible for inclusion of this present study.

### Study Characteristics

Among the 10 included studies, 7 were RCTs,^3–8,18^ 2 were prospective controlled cohorts,^19,20^ and 1 was retrospective controlled cohort.^21^ Five studies were multicenter studies.^3,5,6,8,18^ These studies involved a total of 1682 ACLF patients, among whom 842 were treated with ALSS.

Countries involved in these studies were Germany, China, USA, Spain, UK, Belgium, Italy, Austria, Croatia, Denmark, France, Belgium, and Switzerland. The follow-up periods ranged from 28 days to 5 years. Table 1 described the main characteristics of these studies.

**TABLE 1:**
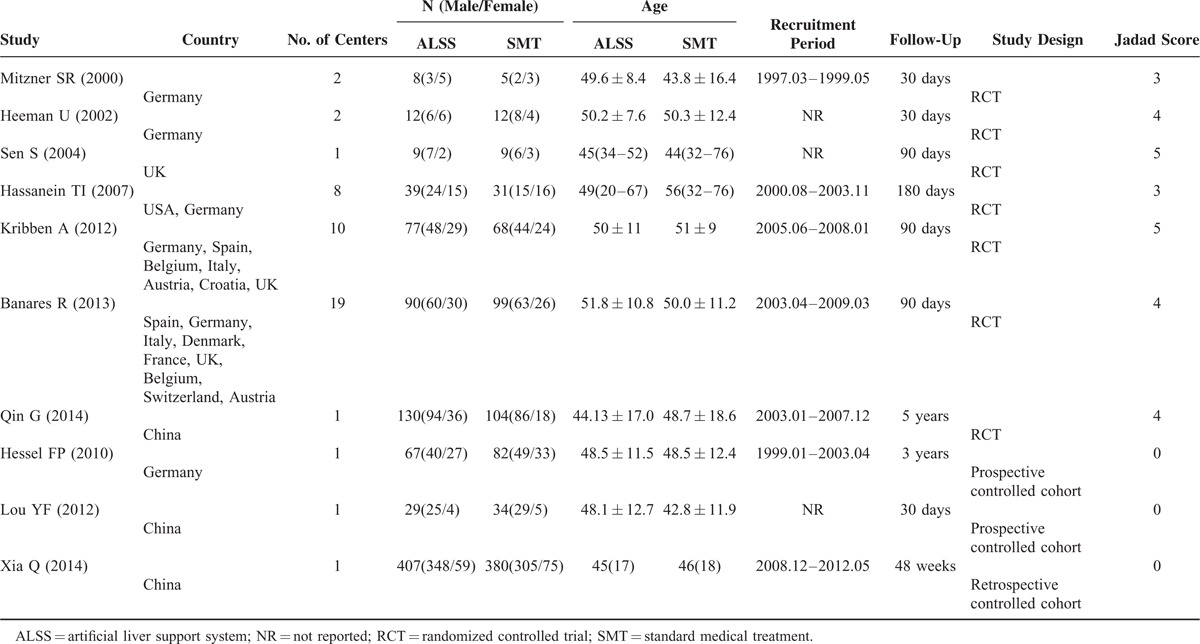
Characteristics of the Included Studies

The definitions of ACLF differed among the studies and were mainly based on the relevant guidelines from Asian Pacific Association for the Study of the Liver (APASL), European Association for the Study of the Liver (EASL), American Association for the Study of Liver Diseases (AASLD), or Chinese Society of Hepatology. The disease etiology of most patients was alcohol ingestion or viral hepatitis. Two studies enrolled patients with ACLF caused solely by HBV reactivation,^7,20^ which was defined as reappearance or increase of serum HBV DNA levels (ie, ≥ 1 log as compared with baseline).^22^ Other studies recruited patients with varied etiology including HCV infection, alcoholic-related, drug-induced, or autoimmune liver diseases (Table s1).

Table s2. showed the inclusion and exclusion criteria of each study. Many aspects of these criteria were in agreement with each other. Only adult (usually aged 18–75 years old) patients were recruited in these studies. Hepatobiliary obstruction, severe acute hemorrhages, malignancies (hepatic/extrahepatic), severe comorbid conditions such as cardiopulmonary diseases, chronic renal insufficiency or diabetes mellitus, and pregnancy were listed in the exclusion criteria in most of the studies.

Treatment characteristics in the included studies were shown in Table s3. All the studies used ALSS in their intervention arm with some variation. Concerning the therapeutic strategy in the treatment group, 3 studies adopted PE,^7,20,21^ 6 studies adopted MARS,^3–6,18,19^ 1 study adopted FPSA.^8^ In some studies, none of the patients were eligible for liver transplant,^5^ or liver transplant was one of the exclusion criteria.^3,19^ In other studies, number of transplant patients were similar between study arms,^6,8,18^ except for 1 study.^21^ Two studies did not report the information on liver transplant.^4,20^

### Association of ALSS With Short-Term Survival in ACLF

**FIGURE 2:**
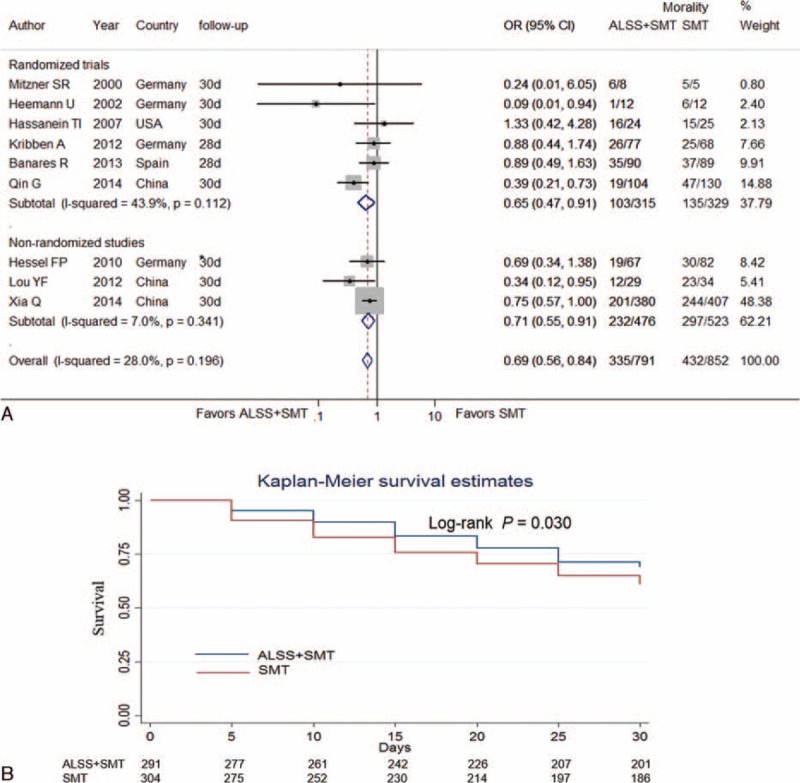
Forest plots showing 1-month mortality in ACLF patients treated with ALSS groups or control groups (A). Kaplan–Meier curve showing pooled 1-month survival in ACLF patients in ALSS groups or control groups (B). ACLF = acute-on-chronic liver failure; ALSS = artificial liver support system; OR = odds ratio; SMT = standard medical treatment. ^∗^ The data were extracted by digitizing graphs using the GetData Graph Digitizer (version 2.24, Russian Federation).

Nine studies reported 1-month mortality of ACLF patients and involved 791 patients in ALSS groups and 852 in the control groups.^3,5–8,18–21^ There were 335 (42.35%) and 432 (50.70%) deaths during first 1 month in ALSS groups and control groups, respectively. The mortality was significant lower in ALSS groups (OR, 0.69; 95% CI, 0.56–0.84 [*P* < 0.001, *I*^*2*^ = 28%]) (Figure 2 A). In the subgroup analysis, 6 randomized trials showed that the association of ALSS with 1-month mortality reduction was significant (OR, 0.65; 95% CI, 0.47–0.91 [*P* = 0.012, *I*^*2*^ = 43.9%]); and the same result was obtained from 3 nonrandomized studies (OR, 0.71; 95% CI, 0.55–0.91 [*P* = 0.007, *I*^*2*^ = 7%]).

Four studies provided 1-month Kaplan–Meier curves of the ACLF patients, comparing ALSS with the control.^7,8,18,19^ The raw data were either obtained from texts^18^ or extracted by digitizing graphs using the GetData software.^7,8,19^ The pooled Kaplan–Meier curve showed that the overall survival rate in ALSS groups was higher than that in the control groups (Log-rank test *P* = 0.03) (Figure 2B).

**FIGURE 3:**
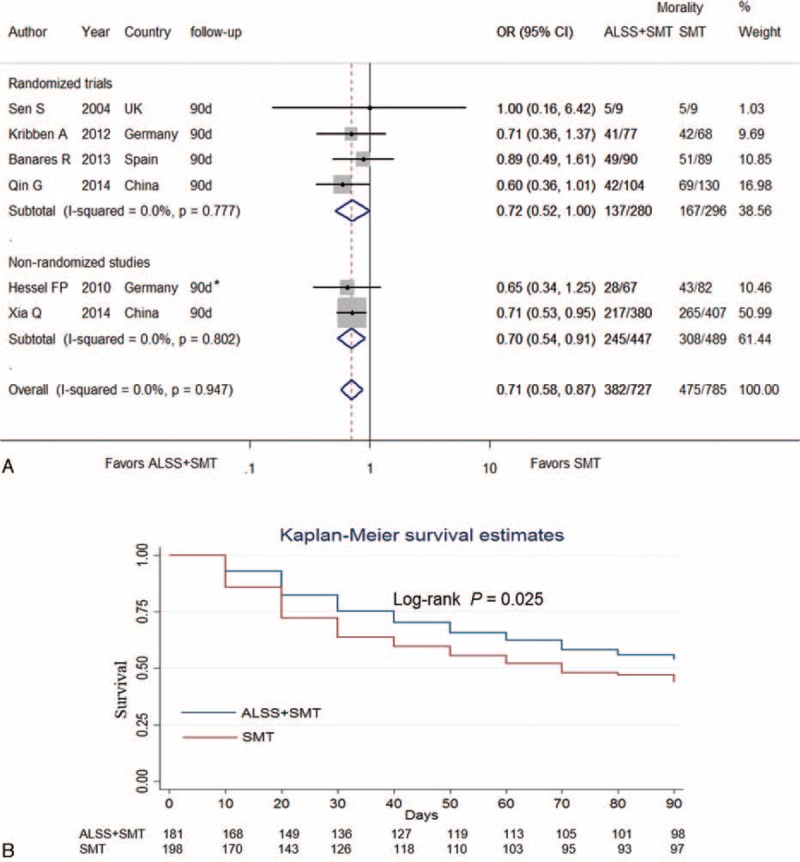
Forest plots showing 3-month mortality in ACLF patients in ALSS groups or control groups (A). Kaplan–Meier curve showing pooled 3-month survival in ACLF patients in ALSS groups or control groups (B). ACLF = acute-on-chronic liver failure; ALSS = artificial liver support system; SMT = standard medical treatment; OR = odds ratio. ^∗^ The data were extracted by digitizing graphs using the GetData Graph Digitizer (version 2.24, Russian Federation).

Six studies reported 3-month mortality and involved 727 patients in ALSS groups and 785 patients in the control groups.^4,7,8,18,19,21^ 382 (52.54%) and 475 (60.51%) died during the first 3 months in ALSS groups and the control groups respectively. Patients in ALSS groups had significantly lower risk of 3-month mortality (OR, 0.71; 95% CI, 0.58–0.87 [*P* = 0.001, *I*^*2*^ = 0%]). In the subgroup analysis, the association of ALSS with 3-month mortality reduction remained significant both in randomized trials (OR, 0.72; 95% CI, 0.52–1.00 [*P* = 0.05, *I*^*2*^ = 0%]) and in nonrandomized studies (OR, 0.70; 95% CI, 0.54–0.91 [*P* = 0.009, *I*^*2*^ = 0%]) (Figure 3 A).

Three studies provided 3-month Kaplan–Meier curves of the ACLF patients, comparing ALSS with SMT.^7,8,19^ The raw data were either obtained from texts^7^ or extracted by digitizing graphs using the GetData software.^8,19^ The pooled Kaplan–Meier curve showed that there was a significant difference in 90-day survival between patients in ALSS groups (Log-rank test *P* = 0.025) (Figure 3B).

### Association of ALSS With Medium-Term Survival in ACLF

**FIGURE 4:**
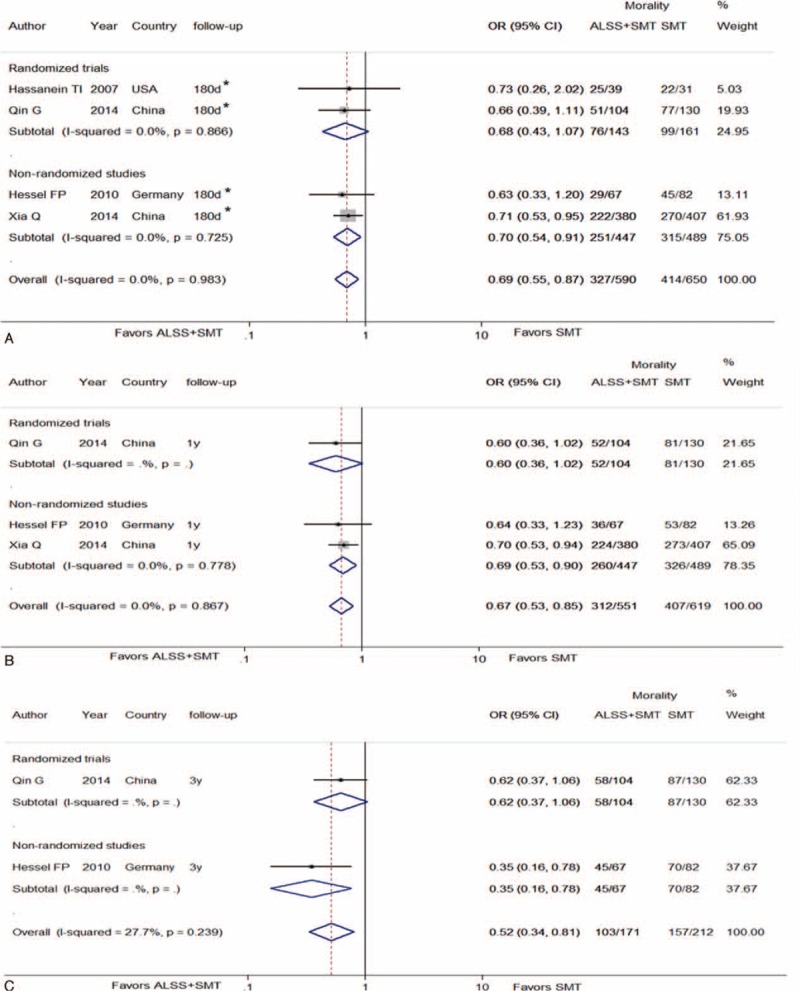
Forest plots showing 6-month (A), 1-year (B), and 3-year (C) mortality in ACLF patients in ALSS groups or control groups. ACLF = acute-on-chronic liver failure; ALSS = artificial liver support system; OR = odds ratio; SMT = standard medical treatment.

Four studies reported 6-month survival rates, comparing ALSS groups with the control groups.^3,7,19,21^ Our meta-analysis indicated significant reductions of the mortality in patients with ALSS therapy (OR, 0.69; 95% CI, 0.55–0.87 [*P* = 0.002, *I*^*2*^ = 0%]). However, in the subgroup analysis, the association of reduced 180-day mortality in the ALSS group remained significant in nonrandomized studies (OR, 0.70; 95% CI, 0.54–0.91 [*P* = 0.008, *I*^*2*^ = 0%]) whereas no significant in randomized trials (OR, 0.68; 95% CI, 0.43–1.07 [*P* = 0.097, *I*^*2*^ = 0%]) (Figure 4A).

Three studies reported 1-year survival rate comparing ALSS with control.^7,19,21^ Our meta-analysis demonstrated that ALSS significantly reduced mortality in ACLF patients (OR, 0.67; 95% CI 0.53–0.85 [*P* = 0.001, *I*^*2*^ = 0%]). With the subgroup analysis, 2 nonrandomized studies suggested that the association for reduction mortality in the ALSS group compared with the SMT group remained significant in (OR, 0.69; 95% CI, 0.53–0.90 [*P* = 0.007, *I*^*2*^ = 0%]), whereas only 1 randomized trial reported an insignificant association (OR, 0.60; 95% CI, 0.36–1.02 [*P* = 0.06, *I*^*2*^ = 0%]) (Figure 4B).

### Association of ALSS With Long-Term Survival in ACLF

Two studies reported 3-year survival rates, comparing ALSS with the control.^7,19^ The meta-analysis showed significant reductions in the mortality following ALSS therapy (OR, 0.52; 95% CI, 0.34–0.81 [*P* = 0.06, *I*^*2*^ = 27.7%]) (Figure 4C).

Only 1 study reported 5-year survival rates, comparing ALSS with the control.^7^ This study suggested a significant reduction in the mortality following ALSS therapy (OR, 0.58; 95% CI, 0.34–1.00 [*P* = 0.049]).

### Adverse Events

Adverse events were reported inconsistently in the included studies. The incidence of 5 serious adverse events (ie, bleeding, hypotension, infection, coagulopathy, and respiratory failure) were reported by at least 2 trials and thus pooled for analysis. The results showed little significant difference between ALSS groups and control groups (Figure s1). Meanwhile, the higher incidence of catheter-related events and skin rash in ALSS-treated patients was reported by Hassanein et al and Qin et al respectively.^3,7^ Other adverse events were summarized in Table s4.

### Meta-Regression and Sensitivity Analysis

We used meta-regression analysis to investigate possible sources of heterogeneity among the included studies (Figure s2). The results indicated that none of the 6 factors (year of publication, country of origin, number of centers, number participants, Jadad scores, or ALSS methods) explained the heterogeneity (*P* > 0.05). According to our meta-regression analysis, difference concerning ALSS methods seems less important as previously acknowledged.^23^

The sensitivity analysis showed that no individual study significantly affected the summarized results of the clinical outcomes (Figure. s3).

### Publication Bias

We evaluated publication bias for the pooled ORs and CIs with Begg's and Egger's tests. The publication bias was *P* < 0.05 in Begg's test and Egger's test, respectively (data not shown). Figure s4, showed the funnel plots.

## DISCUSSION

Acute-on-chronic liver failure is a serious medical ailment and associated with high mortality. Artificial liver support systems have been applied in patients with ACLF for nearly 2 decades. It has no justification to compare ALSS with the artificial renal support system (ARSS). Renal failure (RF) is resulted from intense reduction in the glomerular filtration rate (GFR) and the retaining water-soluble substances can be easily removed with current ARSS technology. In contrast, ACLF is a much more complex syndrome.^2^ Given the unique roles that the liver plays, the functions that artificial liver support devices should perform include: removal of toxins (such as ammonia and aromatic amino acids), replenishment of plasma proteins (such as coagulation factors and albumin), and reversal of the massive inflammatory process initiated from the necrotic liver. Although the current liver support devices do not allow correction of all metabolic disturbances in liver failure, controversies have been focused on the role of ALSS whether as a bridge to liver transplantation (may be dead end street if LT not available) or a bridge to recovery (independent pathfinder). In the present study, we have tried to answer the crucial question about the real impact of these devices on patient survival.

This review of 7 randomized trials and 3 nonrandomized studies compared the effect of artificial liver support system with standard medical therapy for acute-on-chronic liver failure. The meta-analysis found that ALSS reduced the risk of short-term (1-month and 3-month) mortality for patients with ACLF by nearly 30%. Randomized trials and observational studies provided good internal and external validity respectively. The combined Kaplan–Meier curves showed a consistent pattern of findings. In 2 randomized trials, ALSS was found to reduce medium-term (6-month and 1-year) mortality by 30% and long-term (3-year) mortality by 50% in ACLF patients. Actually, the prognosis of ACLF patients may be altered by treatment options other than ALSS. For instance, treatment with nonselective β blockers (NSBBs) may be another factor which might affect the outcome of patients with ACLF.^24^ However, data concerning the treatment variables were quite limited in the included studies. Although our results concerning the long-term effects of ALSS seem to be promising, the conclusion is inclusive. Our study highlights the need for more randomized trials for detailed analyses in the future before ALSS could be recommended as “a bridge to recovery” for routine practice.

It should be noted that the primary endpoints of most included studies were defined as liver transplant-free survival within certain periods. Although liver transplant was not one of the exclusion criteria, the number of transplant patients was comparable between study arms in most studies. Therefore, the effect of ALSS may not be counterbalanced by the treatment of transplant.

Results from our meta-analysis are in consistence with 3 earlier meta-analyses,^9,10,13^ but contrast with 2 other meta-analyses.^11,12^ It should be noted that our study differed from previous reviews in several aspects. First, due to the stringent selection criteria, some trials which had been included in other meta-analysis were excluded.^25,26^ Second, bio-artificial systems,^27–29^ due to their greater complexities, were not included here. Third, latest trial reports were replenished in this study. Two interim reports ^30,31^ in previous reviews were replaced by the final reports of these trials.^8,18^ The largest trial to date of ALSS use in ACLF were added for analysis.^7^ Last but not least, unlike the analysis with mortality rates from different time points in previous studies,^9–13^ we made point-to-point comparison of survival data with the help of Getdata software.

ALSS may be associated with a few serious and nonserious adverse events. However, in serious ailments such as ACLF, it may be difficult to establish the correlation between unwanted events and an intervention. Our analysis revealed no significant increase in risk of bleeding, hypotension, infection, coagulopathy, and respiratory failure. Adverse events were reported inconsistently, these results should be interpreted with caution. Additional studies addressing the safety issues in larger population are required before the definitive conclusions could be drawn.

Admittedly, our study has several limitations. First, the sample sizes of 2 individual trials were small ^5,6^ and may produce false negative or false-positive conclusions due to random error. Fortunately, the remaining 5 trials had adequate sample sizes and described their calculation methods.^3,4,7,8,18^ Second, due to the limited number of RCTs, a few nonrandomized studies were included for analysis. The selection bias and confounding were inevitable in these studies. However, it has been argued that observational studies may have better external validity as patients who are willing to enter a randomized trial differ from those who are not.^32^ Finally, analysis according to disease definition or etiology could not be conducted here. Instead, meta-regression analysis with countries, which may be associated with disease definition and etiology, did not show significant heterogeneity.

In conclusion, this time series based meta-analysis demonstrated that ALSS therapy reduced short-term mortality in patients with ACLF. Meanwhile, its impacts on medium- and long-term survival seem to be promising but remained inconclusive. Besides, ALSS did not appear to increase the risks of main serious adverse events. Clinical utility of the device for survival benefit may be implied.

## Supplementary Material

Supplemental Digital Content
