# Supplementary material for: Survival Benefits With Artificial Liver Support System for Acute-on-Chronic Liver Failure: A Time Series-Based Meta-Analysis
Source: Medicine (Baltimore). 2016 Jan 22;95(3):e2506. doi: 10.1097/MD.0000000000002506 (PMC4998263; doi:10.1097/MD.0000000000002506)
Supplement: Supplemental Digital Content [file medi-95-e2506-s001.pdf]

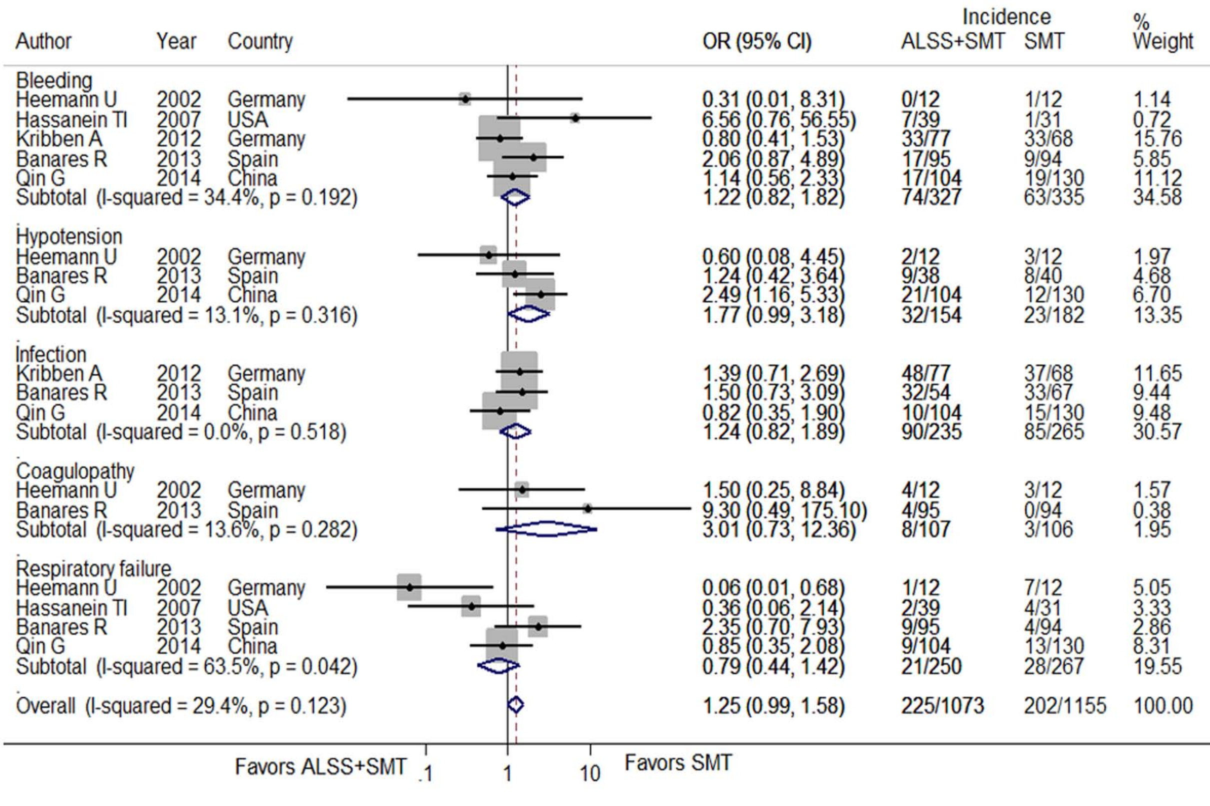
Author Year Country
OR (95% CI)
Incidence
ALSS+SMT SMT
% Weight
Bleeding
Heemann U 2002 Germany 0.31 (0.01, 8.31) 0/12 1/12 1.14
Hassanein TI 2007 USA 6.56 (0.76, 56.55) 7/39 1/31 0.72
Kribben A 2012 Germany 0.80 (0.41, 1.53) 33/77 33/68 15.76
Banares R 2013 Spain 2.06 (0.87, 4.89) 17/95 9/94 5.85
Qin G 2014 China 1.14 (0.56, 2.33) 17/104 19/130 11.12
Subtotal (I-squared = 34.4%, p = 0.192) 1.22 (0.82, 1.82) 74/327 63/335 34.58
Hypotension
Heemann U 2002 Germany 0.60 (0.08, 4.45) 2/12 3/12 1.97
Banares R 2013 Spain 1.24 (0.42, 3.64) 9/38 8/40 4.68
Qin G 2014 China 2.49 (1.16, 5.33) 21/104 12/130 6.70
Subtotal (I-squared = 13.1%, p = 0.316) 1.77 (0.99, 3.18) 32/154 23/182 13.35
Infection
Kribben A 2012 Germany 1.39 (0.71, 2.69) 48/77 37/68 11.65
Banares R 2013 Spain 1.50 (0.73, 3.09) 32/54 33/67 9.44
Qin G 2014 China 0.82 (0.35, 1.90) 10/104 15/130 9.48
Subtotal (I-squared = 0.0%, p = 0.518) 1.24 (0.82, 1.89) 90/235 85/265 30.57
Coagulopathy
Heemann U 2002 Germany 1.50 (0.25, 8.84) 4/12 3/12 1.57
Banares R 2013 Spain 9.30 (0.49, 175.10) 4/95 0/94 0.38
Subtotal (I-squared = 13.6%, p = 0.282) 3.01 (0.73, 12.36) 8/107 3/106 1.95
Respiratory failure
Heemann U 2002 Germany 0.06 (0.01, 0.68) 1/12 7/12 5.05
Hassanein TI 2007 USA 0.36 (0.06, 2.14) 2/39 4/31 3.33
Banares R 2013 Spain 2.35 (0.70, 7.93) 9/95 4/94 2.86
Qin G 2014 China 0.85 (0.35, 2.08) 9/104 13/130 8.31
Subtotal (I-squared = 63.5%, p = 0.042) 0.79 (0.44, 1.42) 21/250 28/267 19.55
Overall (I-squared = 29.4%, p = 0.123) 1.25 (0.99, 1.58) 225/1073 202/1155 100.00
Favors ALSS+SMT
.1
1
10
Favors SMT

Figure s1

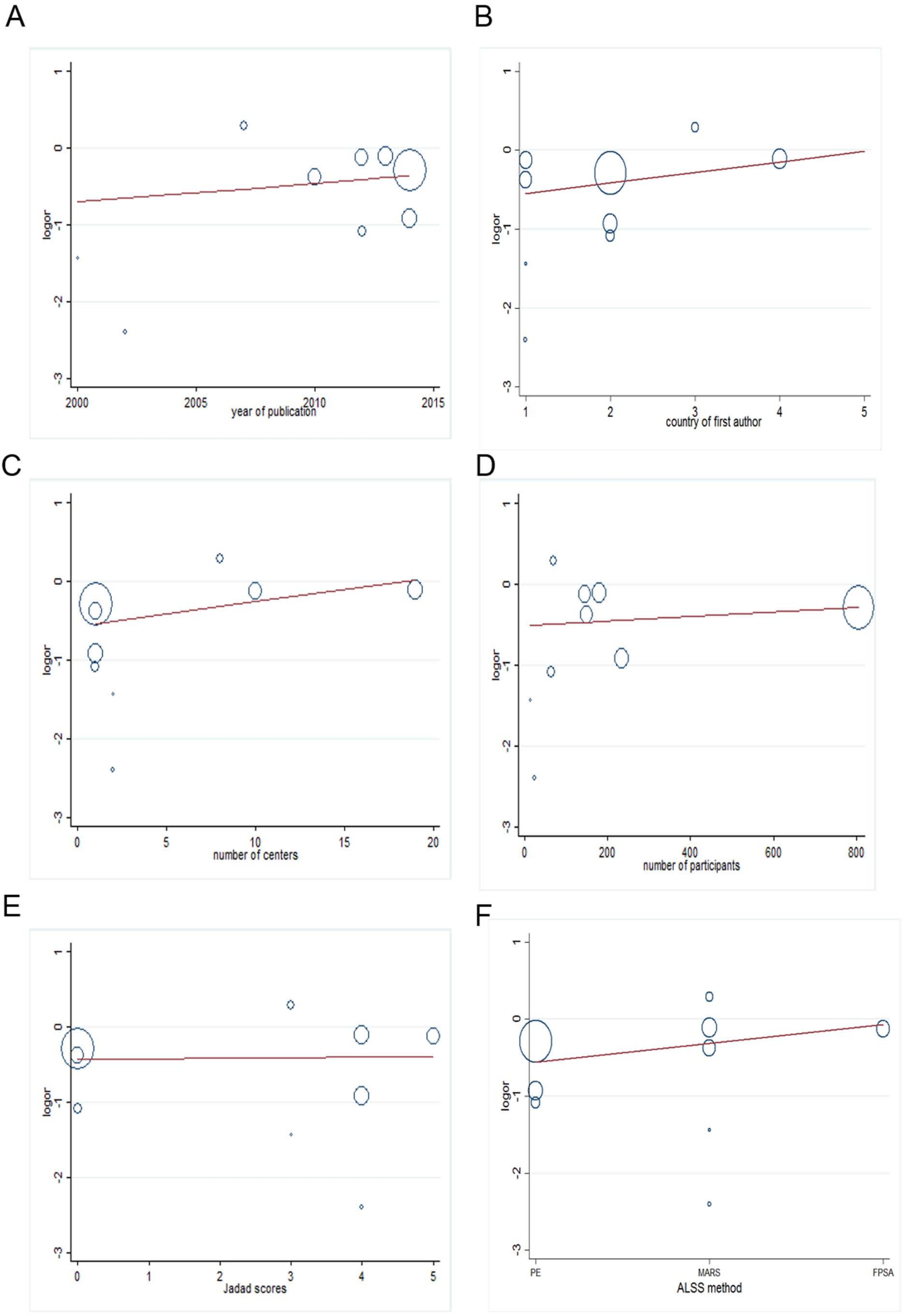
A
logor
year of publication
2000
2005
2010
2015
B
logor
country of first author
C
logor
number of centers
D
logor
number of participants
E
logor
Jadad scores
F
logor
ALSS method
PE
MARS
FPSA

Figure s2

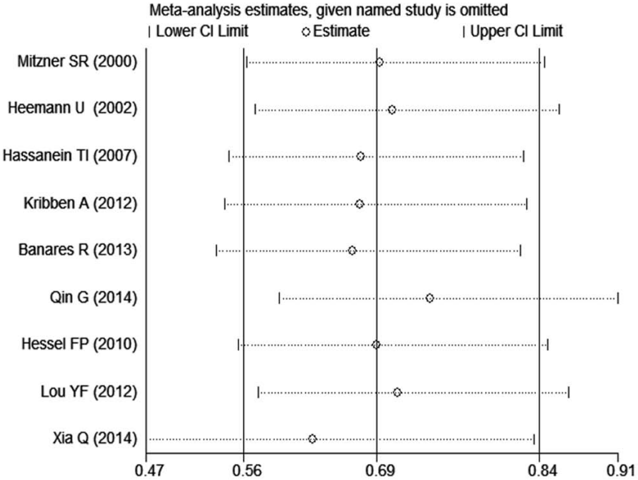
Meta-analysis estimates, given named study is omitted
Lower CI Limit
Estimate
Upper CI Limit
Mitzner SR (2000)
Heemann U (2002)
Hassanein TI (2007)
Kribben A (2012)
Banares R (2013)
Qin G (2014)
Hessel FP (2010)
Lou YF (2012)
Xia Q (2014)
0.47
0.56
0.69
0.84
0.91

Figure s3

A

B

C

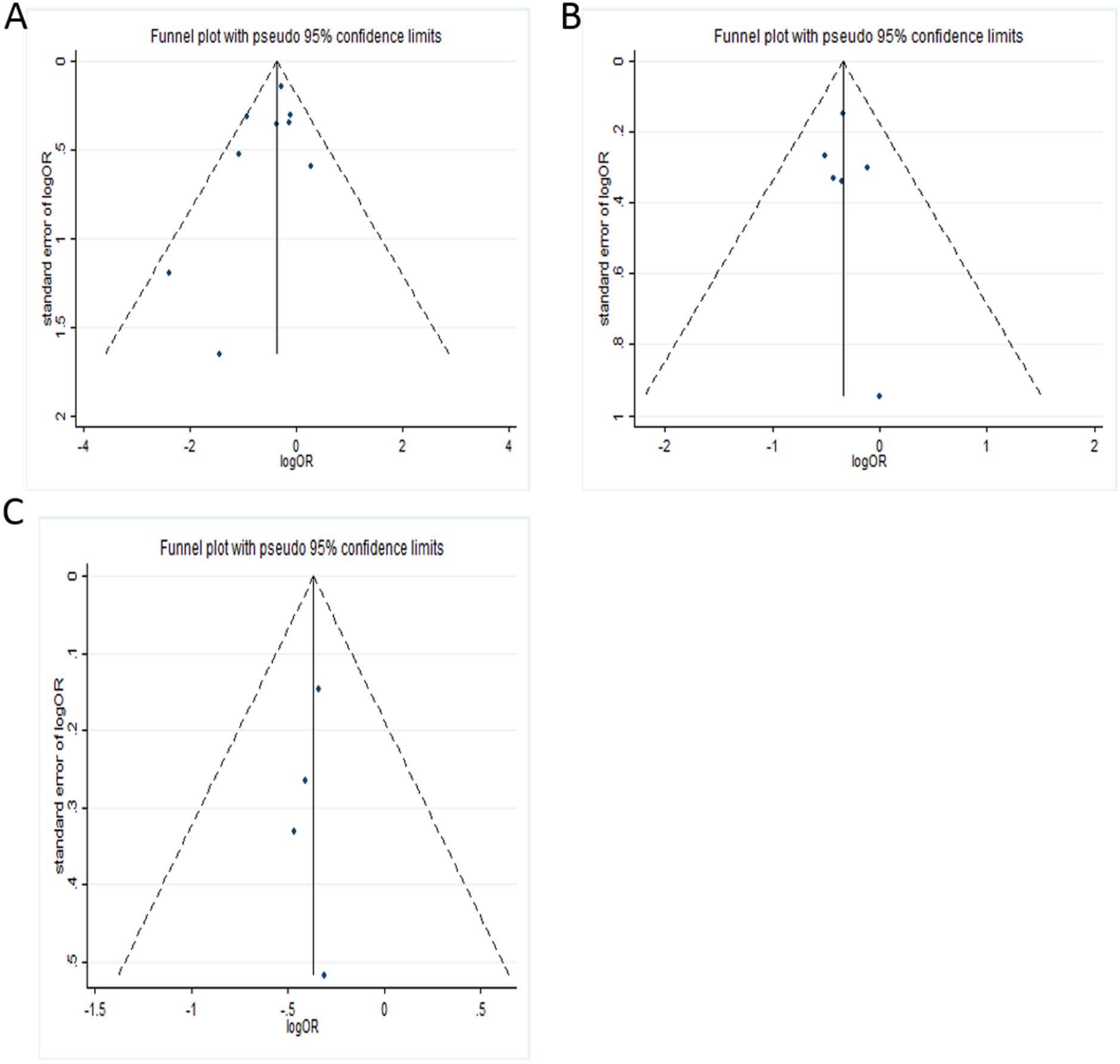

Figure s4

Supplemental TABLES

Table s1. Definition and etiology of ACLF in the included studies

Study	Definition of acute-on-chronic liver failure	Etiology
Mitzner SR (2000)	CTP class C, total bilirubin >15 mg/dL, or HRS requiring hemodialysis.	Alcoholic liver disease, HBV, PBC, secondary biliary cirrhosis
Heeman U (2002)	CTP class B or C, acute decompensation, or total bilirubin >20 mg/dL.	Alcoholic liver disease, HBV, HCV
Sen S (2004)	Acute decompensation over 2-4 weeks, total bilirubin > 5.8 mg/dL, HE grade ≥2, or HRS (no response to SMT for 48 hours).	Alcoholic liver disease
Hassanein TI (2007)	Presumptive diagnosis of cirrhosis, and HE grade ≥3.	Alcoholic liver disease, HBV, HCV, cryptogenic cirrhosis, drug induced, AIH, PSC
Kribben A (2012)	Severe deterioration of chronic liver disease, total bilirubin ≥5 mg/dL and CTP ≥10	Alcoholic liver disease (56%), viral liver disease (20%), alcoholic and viral liver disease (7%), other etiologies (17%)
Banares R (2013)	Presumptive diagnosis of cirrhosis, total bilirubin >5 mg/dL, and at least one of the following: HRS, HE grade 2-4, or total bilirubin >20 mg/dL.	Alcohol (82%), HCV, HBV, AIH, PBC, PSC, NASH, drug toxicity, Wilson disease, etc.
Qin G (2014)	Presumptive diagnosis of CHB, HBV-associated cirrhosis, or HBsAg carrier; total bilirubin >10 mg/dL, within 28 days from symptom onset; INR >1.5 or PTA <40%.	HBV
Hessel FP (2010)	Acute deterioration in liver function over 2-4 weeks, total bilirubin >300 μmol/L, no response to SMT for 48 hours	Alcoholic-related (71%), infections (18%), intoxications (5%), others/unknown (6%)
Lou YF (2012)	Chronic liver disease, positive HBV-DNA with a history of known HBsAg positivity >6 months, total bilirubin >171μmol/L, PTA <40%, HE grade 2-4	HBV

Xia Q(2014)	Acute deterioration of pre-existing chronic liver disease, total bilirubin ≥10 mg/dL or a daily elevation ≥1 mg/dL, PTA ≤40% or INR >1.50.	HBV alone or combined with another cause (91%), alcohol abuse (4%), autoimmune (1%), cholestatic (1%), others (3%)

HRS, hepatorenal syndrome; HE, hepatic encephalopathy; HCV, hepatitis C virus; HBV, hepatitis B virus; AIH, autoimmune hepatitis; PBC, primary biliary cirrhosis; PSC, primary sclerosing cholangitis; NASH, nonalcoholic steatohepatitis; CTP, Child-Turcotte-Pugh; PTA, prothrombin time activity; CHB, chronic hepatitis B; HBsAg, hepatitis B surface antigen; INR, international normalized ratio; PSC, primary sclerosing cholangitis.

Table s2. Inclusion and exclusion criteria in the included studies

Study	Inclusion criteria	Exclusion criteria
Mitzner SR (2000)	(1) Ascites and clinical, biochemical, and ultrasonographic signs of liver cirrhosis;<br>(2) hyperbilirubinemia (TBIL > 15 mg/dL);<br>(3) HE (grade I-IV).	(1) Fulminant hepatic failure;<br>(2) sepsis unresponsive to antibiotic treatment;<br>(3) severe acute hemorrhages;<br>(4) malignancies;<br>(5) obstructive/chronic renal failure;<br>(6) pregnancy;<br>(7) severe cardiopulmonary disease.
Heeman U (2002)	(1) Aged between 18 and 65 years old;<br>(2) cirrhosis (CTP 7 and higher);<br>(3) superimposed acute liver injury leading to decompensation and severe hyperbilirubinemia (TBIL > 20 mg/dL).	(1) Hepatobiliary obstruction;<br>(2) inability to undergo the extracoporeal treatment because of active bleeding or sepsis causing hemodynamic instability;<br>(3) comorbid conditions associated with a poor outcome (as necrotic pancreatitis, cancer or cardiopulmonary failure);<br>(4) coma of nonhepatic origin;<br>(5) extensive surgery during the month before admission;<br>(6) pregnancy.
Sen S (2004)	(1) Acute deterioration in liver function over 2-4 weeks with a defined inflammation-related precipitant (infection or alcoholic hepatitis) leading to severe progressive clinical deterioration despite supportive care (over 48 hours) with increasing jaundice (TBIL > 100 μmol/L);<br>(2) HE (Grade 2) or HRS;<br>(3) clinical, radiological, biochemical, and histological evidence of cirrhosis.	(1) Age < 18 or >75 years;<br>(2) lack of consent/assent;<br>(3) prior enrollment in another study;<br>(4) known hepatic/extrahepatic malignancy;<br>(5) uncontrolled infection or UGIB over the previous 48 hours;<br>(6) pregnancy;<br>(7) prior treatment with terlipressin for HRS,<br>(8) coexisting HIV infection;

		(9) severe cardiorespiratory disease.
Hassanein TI (2007)	(1) 18 years of age or older;<br>(2) cirrhosis and HE grade 3 or 4.	(1) Active hemorrhage;<br>(2) hemodynamic instability;<br>(3) acute cardiopulmonary complications (pulmonary edema, massive aspiration pneumonia, heart failure);<br>(4) pregnancy;<br>(5) active renal replacement therapy;<br>(6) drug intoxication;<br>(7) irreversible brain damage;<br>(8) nonhepatic causes of altered mental status;<br>(9) acute liver failure;<br>(10) hepatocellular carcinoma;<br>(11) liver transplant recipient.
Kribben A (2012)	Severe deterioration of chronic liver disease with TBIL ≥5 mg/dL and CTP ≥10	(1) Circulatory shock;<br>(2) persistent bleeding;<br>(3) INR ≥ 3, or platelet count ≤ 30,000/μL.
Banares R (2013)	(1) Existence of a presumptive diagnosis of cirrhosis;<br>(2) an identifiable triggering event;<br>(3) an increase of TBIL greater than 5 mg/dL;<br>(4) at least one of the following findings: HRS, HE equal or greater than grade II; rapidly progressive hyperbilirubinemia (defined as a more than 50% increase from TBIL levels at admission) greater than 20 mg/dL.	(1) Progressive jaundice as a consequence of the natural course of cirrhosis or extrahepatic cholestasis;<br>(2) platelet count less than 50,000/mm^3;<br>(3) INR >2.3;<br>(4) suspected or evident DIC;<br>(5) need for renal replacement therapy or intrinsic renal disease;<br>(6) uncontrolled infection;<br>(7) active bleeding;<br>(8) HCC >4 cm in diameter or tumoral and nontumoral portal vein thrombosis;

		(9) severe cardiopulmonary disease;<br>(10) hemodynamic instability;<br>(11) major surgical procedure within the last 4 weeks;<br>(12) HIV infection.
Qin G (2014)	(1) Age between 18 and 70 years of age;<br>(2) presumptive diagnosis of CHB, HBV-associated cirrhosis, or HBsAg carrier;<br>(3) rapidly progressive hyperbilirubinemia with TBIL >10 mg/dL, within 28 days from symptom onset;<br>(4) coagulopathy with INR >1.5 or PTA <40%.	(1) Acute HBV infection;<br>(2) superinfection with other viruses (hepatitis E, A, D, or C);<br>(3) superinfection with HIV;<br>(4) other causes of chronic liver failure such as alcohol- or drug-induced liver injury;<br>(5) severe gastrointestinal bleeding;<br>(6) coexistent HCC;<br>(7) pregnancy.
Hessel FP (2010)	Acute deterioration in liver function over 2-4 weeks with a defined inflammation-related precipitant (infection or alcoholic hepatitis) leading to severe progressive clinical, deterioration despite supportive care (over 48 h) with increasing jaundice (≥ 300 μmol/L at least once within a 7 day course) and a hospital stay of more than 6 days.	(1) Severe gastrointestinal bleeding;<br>(2) waiting list for liver transplantation;<br>(3) known carcinoma;<br>(4) other severe comorbidities.
Lou YF (2012)	(1) Significant asthenia, extreme anorexia, vomiting and abdominal distention;<br>(2) rapidly increased blood bilirubin with total bilirubin>171μmol/L;<br>(3) PTA <40%;<br>(4) symptoms of chronic liver disease, including splenomegaly, atrophy of the right lobe along with enlargement of the left lobe and varices or collaterals on ultrasonography or CT;<br>(5) positive HBV-DNA with a history of known HBsAg positivity >6 months	(1) Acute liver failure;<br>(2) graft non-function after liver transplantation;<br>(3) uncontrolled systemic or intracranial bleeding;<br>(4) brain stem herniation;<br>(5) severe hypotension; angiotensin-converting enzyme inhibitors in use and pregnancy. Concurrence of HCV, HDV, HGV, HIV infections;<br>(6) AILD.

	(6) established diagnose of stage II or higher HE.	
Xia Q (2014)	(1) Acute deterioration of pre-existing chronic liver disease;<br>(2) extreme fatigue with severe digestive symptoms, such as obvious anorexia, abdominal distension or nausea and vomiting;<br>(3) TBIL ≥10 mg/dL or a daily elevation ≥1 mg/dL;<br>(4) PTA ≤40% (PT≥18.3 s or INR >1.50).	(1) Significant cardiopulmonary comorbidity;<br>(2) intrinsic renal disease;<br>(3) other comorbities such as diabetes or hypothyroidism;<br>(4) failure to meet the Chinese criteria for ACLF.

TBIL, total bilirubin; HE, hepatic encephalopathy; CTP, Child-Turcotte-Pugh index; HRS, hepatorenal syndrome; UGIB, upper gastrointestinal bleeding; HIV, human immunodeficiency virus; INR, international normalized ratio; DIC, disseminated intravascular coagulation; HCC, hepatocellular carcinoma; CHB, chronic hepatitis B; HBV, hepatitis B virus; HBsAg, hepatitis B surface antigen; HEV, hepatitis E virus; HAV, hepatitis A virus; HDV, hepatitis D virus; HCV, hepatitis C virus; PTA, prothrombin time activity; ACEI, angiotensin-converting enzyme inhibitor; HGV, hepatitis G virus; AILD, autoimmune liver disease; PT, prothrombin time; ACLF, acute-on-chronic liver failure.

Table s3. Treatment characteristics in the included studies

Study	ALSS			Liver transplantation		
	Method	No. of ALSS sessions (Duration per session)	Blood Flow Rate (ml/min)	ALSS group	Control group	*P*
Mitzner SR (2000)	MARS	5.25 ± 3.62 sessions (6-8 h)	2 to 3 mg/kg/min	0/8	0/5	
Heeman U (2002)	MARS	up to 10 treatments (6 h)	200 mL/min.	1/12	1/12	> 0.05
Sen S (2004)	MARS	4 sessions (4 h)	NR	NR	NR	
Hassanein TI (2007)	MARS	2.7±1.5 sessions (4 h)	210 (170-500) mL/min	0/24	0/25	
Kribben A (2012)	FPSA	Mean of 8.1 sessions (5.7±1.3 h)	NR	21/77	13/68	> 0.05
Banares R (2013)	MARS	Up to 10 sessions (6-8 h)	100-250 mL/min	3/90	3/89	> 0.05
Qin G (2014)	PE	average 2 sessions (NR)	25-30mL/min	0/104	2/130	> 0.05
Hessel FP (2010)	MARS	Mean of 8.7 sessions (NR)	NR	0/67	0/82	
Lou YF (2012)	PE	Average of 3.2 sessions (6 h)	25-30mL/min	NR	NR	
Xia Q (2014)	PE	2.65±1.32 sessions (NR)	NR	60/380	9/407	< 0.001

ALSS, artificial liver support system; MARS, molecular adsorbent recirculating system; NR, not reported; FPSA, fractionated plasma separation and adsorption; PE, plasma exchange.

Table s4. Adverse events in ALSS groups vs. control groups in the randomized trials

Study	Adverse events	ALSS group	Control group	*P*
Heemann U (2002)	Worsening of hepatic encephalopathy	0/12	3/12	0.217
	Electrolyte disorders	4/12	10/12	0.036
	New formation of ascites	0/12	1/12	1.000
Hassanein TI (2007)	Neurological	5/39	2/31	0.452
	Gastrointestinal and hepatic	8/39	7/31	1.000
	Cardiovascular	10/39	9/31	0.792
	Hematologic	4/39	2/31	0.687
	Renal	5/39	2/31	0.452
	Systemic	10/39	9/31	0.792
	Catheter related	6/39	0/31	0.030
Kribben A (2012)	Recurrent ascites	39/77	38/68	0.617
Banares R (2013)	Pneumonia	10/37	13/40	0.628
	Death related infection	10/32	9/44	0.298
Qin G (2014)	Skin rash	28/104	9/130	<0.001
	Hyperkalemia	19/104	16/130	0.268
	Pneumonia	10/104	17/130	0.537
